# Achieving sub-wavelength imaging through a flat hyperlens in a modified anodic aluminum oxide template

**DOI:** 10.1038/s41598-020-62243-0

**Published:** 2020-03-24

**Authors:** Chung-Wei Tao, Ta-Jen Yen, Tsung-Yu Huang

**Affiliations:** 1 Department of Materials Science and Engineering, National Tsing Hua University, Hsinchu, 30013 Taiwan, ROC; 2 Department of Materials Engineering, Ming Chi University of Technology, New Taipei City, 24301 Taiwan, ROC

**Keywords:** Metamaterials, Sub-wavelength optics

## Abstract

Constrained by the diffraction limit, a lens can only resolve features larger than half of the incident wavelength owing to the decaying nature of evanescent waves. Several novel devices have been proposed, for example, superlenses and hyperlenses to break this limit. In this work, we present a flat hyperlens composed of silver nanowires embedded in a modified anodic aluminum oxide (AAO) template to demonstrate subwavelength imaging. Measurement conducted by the near-field scanning optical microscope at 633 nm suggests that our proposed flat hyperlens can indeed achieve sub-wavelength imaging with a resolution down to 0.34λ and 0.25λ along two orthogonal directions. Furthermore, to confirm the resolution limit of the flat hyperlens, numerical simulations were performed at the incident wavelengths of 633 and 365 nm, and the corresponding resolution were 0.19λ and 0.3λ, respectively, thus paving a route for sub-wavelength photolithography.

## Introduction

In general, the quality of imaging based on conventional optics is inherently constrained by the diffraction limit, which was quantified in 1873 by Ernst Abbe. Owing to the diffraction limit, the best resolution of an image one can obtain from a lens is approximately half of the incident wavelength. This limitation stems from the fact that evanescent waves that carry detailed information of an object’s smallest features are unable to propagate into far fields with their decaying nature^1–3^. Near-field scanning optical microscopy (NSOM) was invented to tackle such limits. It collects evanescent waves from an object at a distance within a few wavelengths of the surfaces, thus enabling sub-wavelength imaging^2^. Still, this technology may suffer from some insufficiencies, such as low signal-to-noise ratio and long scanning time to image a specimen. Meanwhile, instead of collecting evanescent waves in the near field, some researchers proposed superlenses^4–6^ and hyperlenses^2,3,7^, which can support the propagation of evanescent waves to construct a sub-wavelength image. The concept of superlenses can be dated back to 2000, proposed by Pendry *et al*. and comprehended by a medium with a negative refraction of −1^8^. Within the superlenses, evanescent waves could be exponentially increased instead of decayed, thus promising a greater propagation distance compared to conventional lenses. Furthermore, superlenses were developed and investigated using a thin metallic film with negative permittivity only^9,10^. With excitation of the surface plasmons of a metallic thin film, the lens can prevent evanescent waves from decaying to zero. By contrast, a hyperlens composed of hyperbolic metamaterials (HMMs) that are alternating metal and dielectric layers, or metallic nanowires embedded in a dielectric matrix, possesses hyperbolic iso-frequency curves of type I and type II, respectively^11,12^. These hyperbolic dispersion curves stem from the anisotropic permittivity derived from the effective medium theory and support real propagating ${k}_{\perp }$ vector even with high-${k}_{\parallel }$ components. In addition, curved hyperlenses can separate two point-sources at an image plane based on their geometries; therefore, sub-wavelength imaging can be collected by conventional optical lenses. It is worth mentioning that HMMs require no resonance behavior to resolve sub-wavelength images, thus promising broadband operating frequencies. Finally, HMMs can be used for novel applications such as flat lenses^13^, light concentrators^14,15^ and rainbow trapping^16^.

In short, to approach sub-wavelength imaging, many novel optical devices such as superlenses and hyperlenses have been proposed with different imaging mechanisms. However, disadvantages or insufficiencies exist with each device. For example, narrow operating frequencies and ohmic losses for superlenses, and curved shapes for hyperlenses, limit their potential applications, especially in the field of lithography that requires a flat lens, whereas a subwavelength lithography technique was able to greatly facilitate the development of nanodevice fabrication and semiconductor manufacturing with its promising small feature sizes^17^. Furthermore, compared to type II HMMs, the type I HMMs can not only image very small features of an object but also their general outline (i.e., the type I HMMs can support all 𝑘_∥_ vectors while the type II HMMs support only high 𝑘_∥_ vectors)^11,12,18^. Therefore, in this work, although its fabrication procedure is much more complicated, we adopt an anodic aluminum oxide (AAO) template with electroplated silver nanowires to fabricate the hyperbolic metamaterials as a flat hyperlens. It is worth mentioning that we have modified the conventional AAO process to achieve a sub-wavelength patterned gold mask on top of a free-standing AAO template to avoid any transfer process. Finally, enhanced image resolution with the proposed hyperlens was examined by NSOM.

## Design, Fabrication, and Measurement

**Figure 1:**
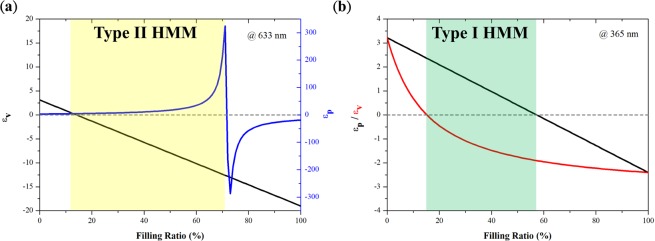
Anisotropic permittivity (ε_p_ and ε_v_) plotted against the filling ratio of metal at wavelengths of (**a**) 633 and (**b**) 365 nm.

To achieve a hyperbolic metamaterial via modified AAO templates, we first determine the corresponding filling ratios of dielectric materials and metal, respectively. According to refs. ^18–20^, the effective permittivity tensor of nanowire-based HMMs is

$${\varepsilon }_{p}=f{\varepsilon }_{m}+(1-f){\varepsilon }_{d},\,and\,{\varepsilon }_{v}=\frac{[(1+f){\varepsilon }_{m}+(1-f){\varepsilon }_{d}]{\varepsilon }_{d}}{(1-f){\varepsilon }_{m}+(1+f){\varepsilon }_{d}}$$

where *f* is the filling ratio of metal; *ε*_*m*_ and *ε*_*d*_ are permittivity of metal and dielectric, respectively; and *ε*_*p*_ and *ε*_*v*_ denote the effective permittivity along the directions parallel and perpendicular to nanowires, respectively. In the AAO template, silver and alumina are used for their dielectric constants of *−19* + *0.53i* and *3.118* at the incident wavelength of 633 nm^21,22^. The corresponding *ε*_*p*_ and *ε*_*v*_ with respect to the filling ratio *f* is portrayed in Fig. 1(a). From the figure, the signs of the two parameters are opposite to each other within the filling ratio from 0.15 to 0.7. By contrast, the dielectric constants of silver and alumina at the incident wavelength of 365 nm, are *−2.4* + *0.25i* and *3.217*, respectively^2,23^. The corresponding *ε*_*p*_ and *ε*_*v*_ with respect to the filling ratio is portrayed in Fig. 1(b). In this case, the signs of the two parameters are opposite to each other within the filling ratio from 0.15 to 0.55. Thus, in summary, the proposed hyperlens could be operated at 633 and 365 nm once the filling ratio of the metal falls in the hyperlens ranges from 0.15 to 0.55. It is worth mentioning that the proposed hyperlens behaves as a type II HMM (i.e., ${\varepsilon }_{p} < 0$ and ${\varepsilon }_{v} > 0$) at the wavelength of 633 nm while acting as a type I HMM (i.e., ${\varepsilon }_{p} > 0$ and ${\varepsilon }_{v} < 0$) at the wavelength of 365 nm. Notably, the effective medium theory may not be applicable to the entire k space, but the proposed hyperlens can still support the propagation of high-${k}_{\parallel }$ waves when the spatial effect is considered^24^.

**Figure 2:**
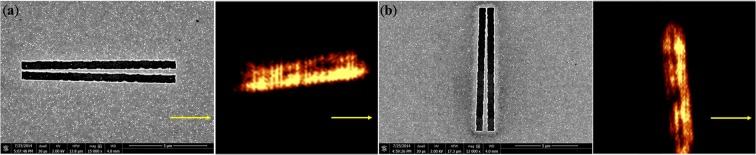
SEM and NSOM images of the horizontal slits on the flat hyperlens.

Meanwhile, to realize the flat hyperlens in experiments, we have adopted the idea of a free-standing nitride film. We modified the original AAO fabrication process into a free-standing AAO template, fabricated through a two-step anodization of aluminum foil^25,26^ and the application of nail polish, to accommodate the following measurement and future possible sub-wavelength lithography process. We fabricated an AAO template 10 μm thick with nanoholes of 60 nm in diameter. Nail polish was applied on both sides with a square open area on the bottom of the AAO template, and a subsequent etching process facilitated the fabrication procedure. Silver, due to its better conductivity in the optical range, was then electroplated into the nanoholes. Note that the geometry of the modified AAO is arbitrarily chosen to meet the required hyperlens filling ratio of approximately 27%. To test the 2-dimensional resolution of our proposed hyperlenshorizontal and vertical slits were inscribed on a gold layer as shown in Fig. 2. Note that one of the slits in each configuration was tilted by a small angle to achieve gradually varied separation for the resolution characterization (see Method for detailed fabrication procedure).

**Figure 3:**
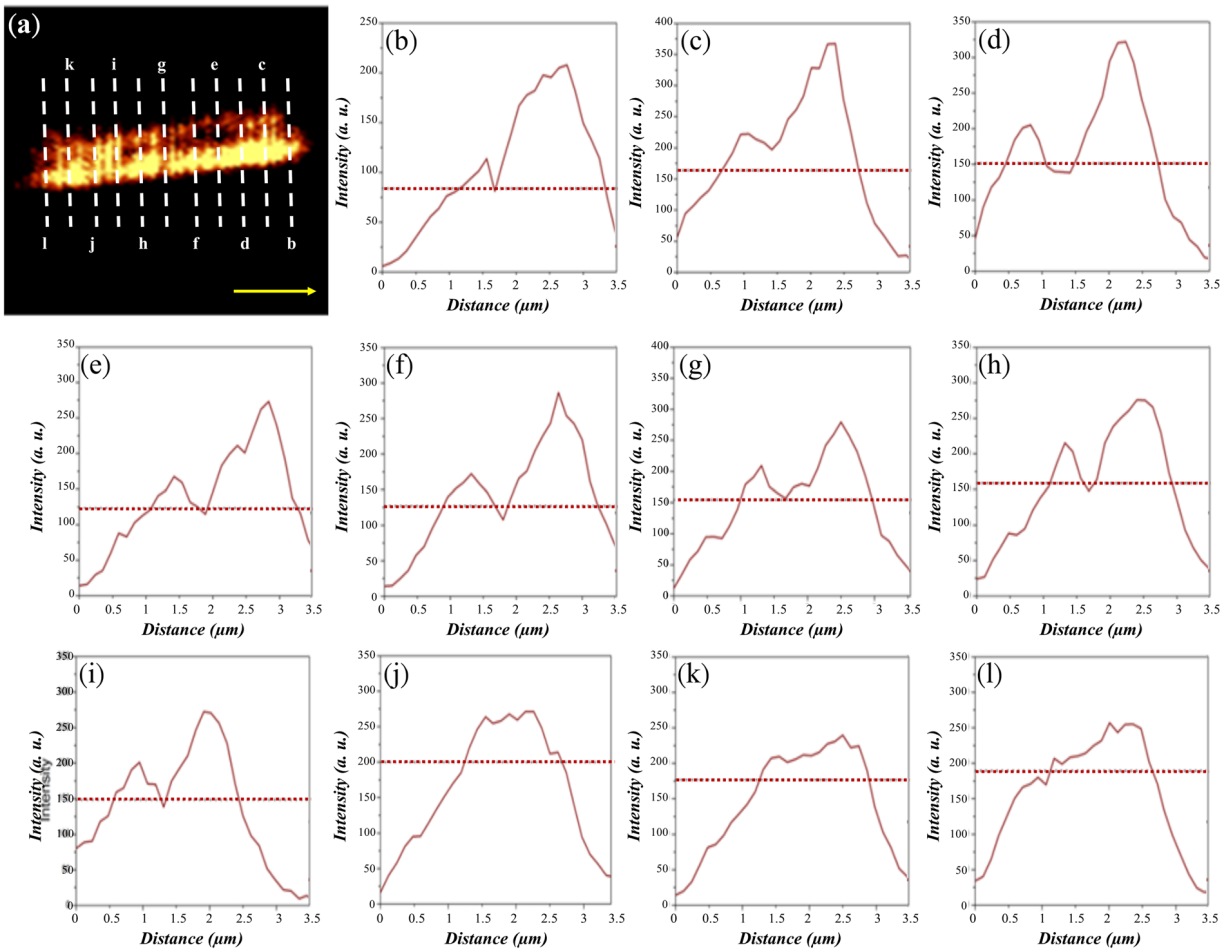
(**a**) Measured NSOM image of the two slits on top of the hyperlens. (**b–l**) Intensity profiles of the NSOM image of the horizontal slits at the different selected lines. From (**i**), the minimum resolution is 0.34λ with a separation of 213.4 nm.

**Figure 4:**
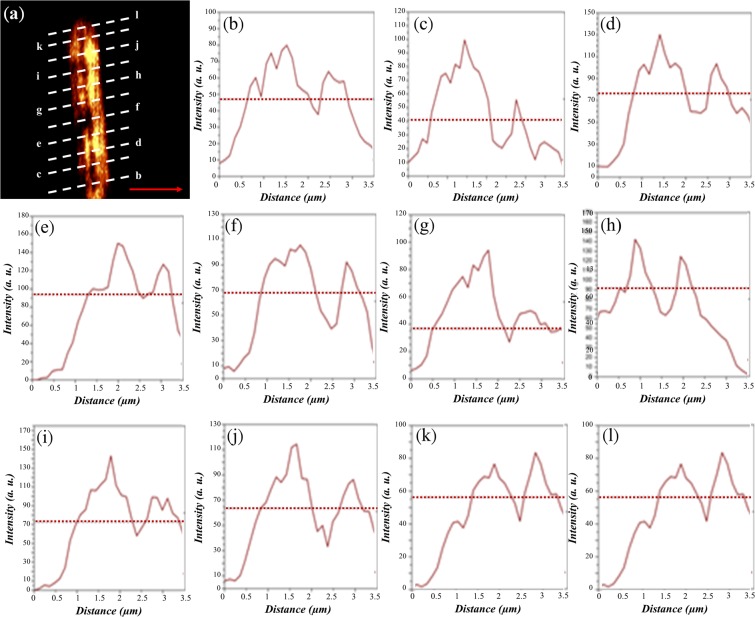
(**a**) Measured NSOM image of the two slits on top of the hyperlens. (**b–l**) Intensity profiles of the NSOM image of the vertical slits at the different selected lines. From (**k**), the minimum resolution is 0.25λ with a separation of 160 nm.

To confirm the resolution provided by the flat lens, NSOM captured the image of the inscribed slits and determined the corresponding resolution along two orthogonal directions. Limited by our light source, only the incident wavelength of 633 nm was used for NSOM measurement under transmission mode to examine the resolution, as illustrated in Fig. 2(a,b) for the horizontal and vertical slits, respectively. From these two figures, we confirmed that some parts of the two slits could be distinguished and resolved in the image plane. To further investigate the resolution of the proposed flat hyperlens, the intensity distribution along the direction perpendicular to one of the slits was analyzed with eleven cutting lines, as shown in Figs. 3 and 4 for the two sets of slits. From these eleven cutting lines, we can determine the corresponding resolution achieved by our proposed hyperlens based on Rayleigh’s criterion, which states that two light sources can be resolved if there is a 26.3% dip between two intensity peaks in the intensity profile^27^.

**Figure 5:**
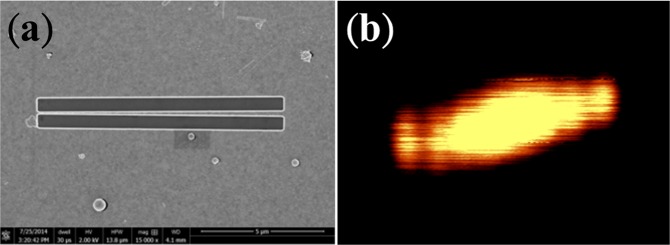
(**a**) SEM image of the inscribed slits on top of a glass substrate. (**b**) Measured NSOM image of the two slits on top of the glass substrate. Note that the probe is lifted from the surface of the imaging objects with a distance equal to the thickness of the flat lens.

Rayleigh’s criterion is shown by the horizontal dashed lines in both Figs. 3 and 4. Therefore, once there appears a dip below the Rayleigh’s criterion between the two intensity peaks, we can claim that the proposed hyperlens can resolve such a separation of the two inscribed slits. Thus, from Fig. 3(i), the lowest resolution achieved by the flat lens is at least 0.34λ with a separation of 213.4 nm between the two slits. Unfortunately, we cannot resolve the two peaks for Fig. 3(c,e) with a separation larger than the expected resolution, which may result from the uneven surface of the AAO template, because the root mean square value of roughness is found to be 10.24 nm, caused by the imperfectly electroplated silver nanowires. We also investigated the resolution for the orthogonal directions. From Fig. 4(b to k), we can observe a dip below the Rayleigh’s criteria for each figure, thus evidencing that the resolution is around 0.25λ with a separation of 160 nm. It is worth stating that the two slits with subwavelength separation could be resolved in the two orthogonal directions at the fixed polarization, indicating that the proposed flat hyperlens could achieve a two-dimensional super resolution. As a comparison, a pair of slits inscribed on the gold layer above the glass substrate was measured by the NSOM system, and the obtained image is illustrated in Fig. 5. Note that the scanning tip is lifted away from the surface of the slits with a distance equal to the hyperlens. The NOSM measurement results indicate that a super-resolution cannot be achieved without the hyperlens because only a merged image can be observed. Notably, in this study, we only arbitrarily chose the fabrication parameters to approach the required filling ratio. Future optimizations may be required to improve the resolution of the proposed hyperlens.

## Simulation Verification

**Figure 6:**
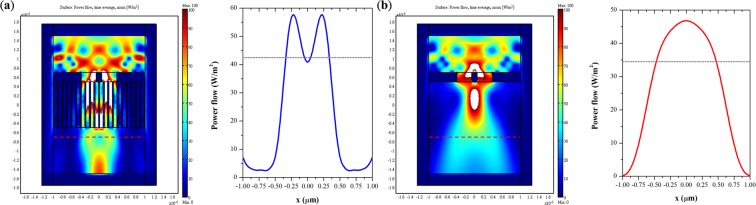
(**a**) Power flow distribution of the hyperlens and the two slits inscribed on a gold layer at 633 nm. The separation distance is 120 nm. The dashed line indicates the imaging plane. Inset shows the power distribution along the dashed line, and the dotted line indicates Rayleigh’s criterion. (**b**) Power flow distribution of free space and the two slits on the gold layer.

**Figure 7:**
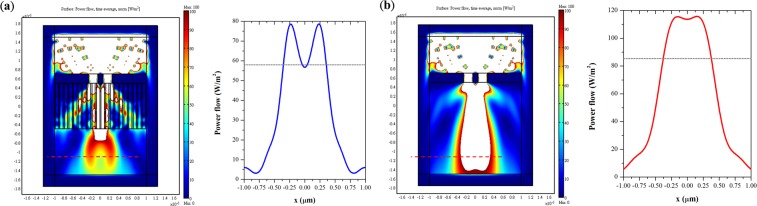
(**a**) Power flow distribution of the hyperlens and the two slits inscribed on a gold layer at 365 nm. The separation distance is 110 nm. The dashed line indicates the imaging plane. Inset shows the power distribution along the dashed line, and the dotted line indicates Rayleigh’s criterion. (**b**) Power flow distribution of free space and the two slits on the gold layer.

To exploit the subwavelength-imaging ability of our proposed hyperlens, we employed a finite element method to demonstrate the theoretical resolution. Note that, to simplify the simulation, we only consider 2D configurations of the HMM flat lens with a filling ratio of 0.27 and reduce the thickness of the lens to 1 μm. A simulation configuration scheme is illustrated in Figs. 6 and 7. A gold layer inscribed with two slits at separation distances of 120 nm and 110 nm function as two line-sources for 633 and 365 nm incident wavelengths, respectively. Below the gold layer is our proposed 1-μm-thick HMM. We monitored the corresponding power flow at image planes 200 and 600 nm away from the bottom of the hyperlens for 633 and 365 nm incident wavelengths, respectively. Two reference configurations are also illustrated and compared in Figs. 6(b) and 7(b) with the identical gold layer on top of 1-μm-thick air. From the simulation, the resolutions of the HMM at the incident wavelengths of 633 and 365 nm are at least λ/5.28 and λ/3.32, respectively. Note that the resolution could be even higher if we replaced air with another high dielectric constant material, for example, photoresist, in simulation. Furthermore, the discrepancy between the experimental measurement and the simulated results might stem from the imperfectly fabricated sample and deviation of the constitutive parameters between simulations and experiments. As a comparison, we also recorded the power flow distribution of the two inscribed slits within the free space, as shown in the insets of Figs. 6(b) and 7(b) for 633 and 365 nm, respectively. It is worth mentioning that these simulation results suggest the optimal resolution based on the perfect periodic AAO templates.

## Conclusion

With the abovementioned theoretical calculation, simulation, and experimental results, we have presented a flat hyperlens composed of silver nanowires embedded in a modified AAO template. We have successfully demonstrated two-dimensional super-resolution with resolutions down to λ/3 and λ/4 in experiments at 633 nm that are beyond the diffraction limit in the two orthogonal directions after a propagation distance of 10 μm through the hyperlens measured by the NSOM system. We also simulated the proposed hyperlens that promises resolutions of approximately 0.19λ and 0.30λ at 633 and 365 nm, respectively. Compared to the concurrent works, for example, non-flat hyperlenses with a resolution of 0.39λ and a magnification of 2.97 under illumination of 410 nm^28^ and the flat hyperlens-based transformation optics with a resolution of 0.1λ but only in simulation^13^, our proposed flat hyperlens reveals better experimental resolution with compatibility to lithography process and could be realized by the modified AAO templates. The flat hyperlens exhibits opposite signs of permittivity along two different axes, i.e., the transverse and vertical directions, and possesses a hyperbolic iso-frequency contour. Furthermore, we can tailor the corresponding permittivity tensor by simply manipulating the filling ratio through controlling the sizes of nanoholes in the modified AAO template. With the aid of our proposed flat hyperlens, a sub-wavelength photolithography can be introduced, paving a route to sub-wavelength-scale optical devices, with the potential to have a significant impact on the fields of electronics, optics, and photonics.

## Method: Fabrication of a Flat Hyperlens

An aluminum foil with high purity (99.997%, provided by Alfa Aesar) was chemically polished in a mixture of ethanol and perchloric acid at 4 °C with an applied voltage of 40 V. Then, a two-step anodization process was conducted. The foil was anodized in 0.3 M oxalic acid with an applied voltage of 40 V in a water bath at 10 °C and a 30-minute reaction time. Because the first anodized alumina is not smooth enough, a removal step was carried out by dipping the sample into a mixture of phosphoric acid, chromic acid, and deionized water. Finally, the second anodization process was conducted for 60 minutes to achieve an AAO template with 10 μm thickness. Typically, diameters of nanoholes on the AAO template ranged from 25 to 35 nm, with an average diameter of 32 nm. They were then expanded to about 54 nm by immersing the template into phosphoric acid for 75 minutes to achieve a filling ratio of 0.266.

Next, 10-nm-thick titanium and 200-nm-thick gold were deposited by E-gun on the sample as adhesion and conduction layers, respectively, for the electroplating process. Note that the imaging objects were also inscribed on this thin gold film for the resolution characterization. Meanwhile, to achieve a free-standing hyperlens, we applied nail polish to the front-side of the sample after deposition. Similarly, we applied nail polish to the back-side of the sample except for a hollow square area. The opening facilitated the etching of the aluminum foil, thus creating the free-standing AAO template. Furthermore, to initiate the electroplating process, native alumina, the aluminum substrate, and the barrier layer were etched out. Alumina was etched by a 1 M sodium hydroxide solution, while aluminum was etched by hydrochloric and copper chloride. Finally, the sample was dipped into a mixture of 5% phosphoric acid to remove the barrier layer.

To electroplate silver onto the AAO template, a potentiostat was employed, and its working and counter electrodes were connected to the AAO template and graphite rod, respectively with the Ag/AgCl/KCl reference electrode. The electrolyte is a mixture of 0.18 M silver nitrate and 0.49 M boric acid. Pulsed electroplating was conducted to achieve uniform silver nanowires with ‘on’ voltage of 0.1 V for 1 s and ‘off’ voltage of 0 V for 5 s. Note that it is necessary to remove overgrown silver outside the AAO template by dipping the sample into nitric acid. Repeated electroplating silver nanowires and removing overgrowth silver nanoparticles could promise a better silver nanowire coverage within the AAO template.

To perform the NSOM measurement, two imaging objects, i.e., horizontal and vertical slits that are composed of two hollow bars are inscribed on top of the gold thin film by focus ion-beam milling. It is worth stating that one of the two bars is tilted by a small angle to achieve gradually varied separation for both horizontal and vertical slits. The width and the length of the hollow bars are 10 μm and 500 nm with a separation ranging from 150 to 400 nm.
